# Strategies for Improving Access to Cancer Services in Rural Communities: A Pre-implementation Study

**DOI:** 10.3389/frhs.2022.818519

**Published:** 2022-03-14

**Authors:** Christina Crabtree-Ide, Nick Sevdalis, Patricia Bellohusen, Louis S. Constine, Fergal Fleming, David Holub, Irfan Rizvi, Jennifer Rodriguez, Michelle Shayne, Nancy Termer, Ken Tomaszewski, Katia Noyes

**Affiliations:** ^1^Department of Cancer Prevention and Control, Roswell Park Comprehensive Cancer Center, Buffalo, NY, United States; ^2^Center for Implementation Science, King's College London, London, United Kingdom; ^3^Judy DiMarzo Cancer Survivorship Program, University of Rochester, Rochester, NY, United States; ^4^Department of Radiation Oncology, Wilmot Cancer Institute, Rochester, NY, United States; ^5^Surgical Health Outcomes & Research Enterprise (SHORE), University of Rochester Medical Center, Rochester, NY, United States; ^6^Department of Family Medicine, University of Rochester, Rochester, NY, United States; ^7^Mid-Atlantic Permanente Medical Group, McLean, VA, United States; ^8^Livingston County Public Health Department, Mt. Morris, NY, United States; ^9^Flatiron Healthcare Inc., New York, NY, United States; ^10^KJT Group, Honeoye Falls, NY, United States; ^11^Department of Epidemiology and Environmental Health, University at Buffalo, Buffalo, NY, United States

**Keywords:** pre-implementation evaluation, multidisciplinary teams, rural health, workforce shortages, community-based participatory research, stakeholder engagement

## Abstract

**Background:**

Implementation science is defined as the scientific study of methods and strategies that facilitate the uptake of evidence-based practice into regular use by practitioners. Failure of implementation is more common in resource-limited settings and may contribute to health disparities between rural and urban communities. In this pre-implementation study, we aimed to (1) evaluate barriers and facilitators for implementation of guideline-concordant healthcare services for cancer patients in rural communities in Upstate New York and (2) identify key strategies for successful implementation of cancer services and supportive programs in resource-poor settings.

**Methods:**

The mixed methods study was guided by the Consolidated Framework for Implementation Research (CFIR). Using engagement approaches from Community-Based Participatory Research, we collected qualitative and quantitative data to assess barriers and facilitators to implementation of rural cancer survivorship services (three focus groups, *n* = 43, survey *n* = 120). Information was collected using both in-person and web-based approaches and assessed attitude and preferences for various models of cancer care organization and delivery in rural communities. Stakeholders included cancer survivors, their families and caregivers, local public services administrators, health providers, and allied health-care professionals from rural and remote communities in Upstate New York. Data was analyzed using grounded theory.

**Results:**

Responders reported preferences for cross-region team-based cancer care delivery and emphasized the importance of connecting local providers with cancer care networks and multidisciplinary teams at large urban cancer centers. The main reported barriers to rural cancer program implementation included regional variation in infrastructure and services delivery practices, inadequate number of providers/specialists, lack of integration among oncology, primary care and supportive services within the regions, and misalignment between clinical guideline recommendations and current reimbursement policies.

**Conclusions:**

Our findings revealed a unique combination of community, socio-economic, financial, and workforce barriers to implementation of guideline-concordant healthcare services for cancer patients in rural communities. One strategy to overcome these barriers is to improve provider cross-region collaboration and care coordination by means of teamwork and facilitation. Augmenting implementation framework with provider team-building strategies across and within regions could improve rural provider confidence and performance, minimize chances of implementation failure, and improve continuity of care for cancer patients living in rural areas.

## Introduction

The rural-urban gap in cancer outcomes continues to expand despite overall improvement in cancer screening and advances in cancer treatments (1, 2). Rural cancer patients and those living in remote regions in the US are significantly more likely to experience problems with accessing health care and have worse health outcomes when compared to non-rural populations (3–5). While cancer puts a heavy physical, emotional, and financial burden on all patients, rural patients are faced with an additional constellation of challenges related to the of their geography and limited access to healthcare (6–8). Access to clinical specialists and multidisciplinary care systems is limited in rural areas of the US where most clinical providers operate solo practices and do not specialize (9–11). As a result, a large proportion of rural cancer patients do not receive coordinated multidisciplinary team-based cancer care recommended by the clinical care guidelines (12–14).

Guideline-concordant multidisciplinary team-based cancer care is defined as the cooperation between different specialized professionals involved in cancer care with the overarching goal of improving treatment efficiency and patient care (15). Examples of multidisciplinary team-based care in oncology, like multidisciplinary tumor boards, and integrative oncology or cancer survivorship programs, requires comprehensive coordination and ongoing communication among many specialists, often across several institutions, for the entire duration of patient cancer care journey. Multidisciplinary cancer care teams may include primary care providers, oncologists, and numerous cancer and ancillary specialists (12, 13, 16, 17). While multidisciplinary cancer care teams have become a norm at large academic cancer centers, rural and small free-standing community oncology clinics rarely possess the resources and personnel to form multidisciplinary teams within and across the regions they serve. Lack of access to guideline-concordant multidisciplinary team-based cancer care among rural cancer patients has been identified as one of the main reasons for rural-urban disparities in cancer outcomes in the US (18, 19).

In the last 20 years, many new clinical and public health programs have been developed to improve quality and continuity of care for geographically isolated populations; however the impact of these programs has been mixed (20). Although several models for comprehensive survivorship programs have been proposed and tested in community oncology settings, little has been reported on how those programs got implemented, what training in survivorship care was provided to the program staff, and which, if any, of numerous recommended survivorship services from Survivorship Care Plans were timely received by rural patients. Across many medical specialties, limited evidence is available to guide the selection of appropriate implementation strategies to tailor guideline recommendations for underserved and under-resourced settings. The Expert Recommendations for Implementing Change (ERIC) project have resulted in nine thematic clusters of the 73 discrete implementation strategies that may be useful for organizing related strategies across studies (21). Examples of implementation strategies include training, remainders, multidisciplinary huddles, IT-based reinforcement, financial incentives, peer support, bottom up and top down strategies, and many others. However, the vast majority of these strategies were tested and developed in the context of large regional health care organizations or public health agencies and may not be suitable in staff and resources limited communities. Differences in staffing, technology, financing, and leadership between rural clinics and large urban care centers pose additional challenges for adaptation, implementation and dissemination of evidence-based practices into rural communities (22–24).

Guided by the Consolidated Framework for Implementation Research (CFIR), this pre-implementation study aimed to identify specific barriers and facilitators to implementing guideline-concordant multidisciplinary cancer services in rural communities of Upstate New York and propose targeted evidence-based implementation strategies consistent with our findings (25). The CFIR is a determinant framework, designed to guide integration of research evidence into practice, adoption, and implementation of evidence-based interventions (e.g., cancer survivorship programs developed for large urban academic cancer centers) by defining social, behavioral, and economic factors that facilitate or impede implementation (e.g., unique characteristics of rural counties and populations). The study was designed to account for the “real-world” complexity of a regional program implementation (limited resources, competing priorities, geographic variation in staffing availability and stakeholder preferences, among others), with a specific focus on potential for scale-up and expansion.

## Methods

### Overview of the Study Approach

This study was conducted as a part of the pre- implementation phases of a community engagement initiative entitled “Virtual Rural Oncology Community” (V-ROC) (26–29). The study took place between January 2016 and December 2017 in rural Finger Lakes counties of New York state. The overarching goals of the V-ROC were (1) to assess barriers and facilitators to guideline-concordant cancer care for rural patients and (2) identify strategies for more efficient implementation and sustainability of guideline-recommended cancer programs in rural communities. The study was guided by the stakeholder engagement principles developed by community-based participatory research (CBPR) (30–32). We define CBPR as a type of community-engaged research that actively involves community stakeholders throughout the research process—from identifying the study questions to selecting research approaches, interpreting results, and disseminating findings. The use of CBPR could be especially critical when working with populations who have a long history of health disparities or mistrust toward clinical research such as rural communities. Use of CBPR methods have been shown to enhance the success of interventions within a real world setting (33).

### Participants

Guided by the CBPR engagement methods, the study team identified of the initial key informants, community advisors and thought leaders (*n* = 5) from one rural county (phase I). The key informants (a primary care physician, a rural surgical oncologist, a nurse, a patient, and a rural county health commissioner) worked together with the study team to identify and recruit a broader patient and provider participants panel from the same county (Livingston County, NY) using snowball approach (34) (phase II). Subsequently, the process was repeated in three other rural counties. The V-ROC cancer stakeholder panel included healthcare professionals, cancer survivors, their families and caregivers, and county health department staff. Health professionals included primary care providers, medical and surgical oncologists, and advanced practice providers (APPs; these are registered nurses and physician assistants) working in a variety of clinical settings, as well as care managers and practice administrators.

The University at Buffalo Institutional Review Board reviewed the study and made a Not Human Research determination because the study questions were focused on healthcare system and services delivery, not participant's health (UB IRB STUDY00004348). Correspondingly, written informed consent from participants was not required to participate in this study.

### Theoretical Framework

The design of V-ROC project was informed by the CBPR approach to allow maximum participation of community stakeholders in all phases of the study. The study data collection tools (interview and focus group guides and surveys) were guided by the Consolidated Framework for Implementation Research (CFIR) (25) to identify the WHAT, WHO, HOW, and WHERE factors that could facilitate or impede implementation of community cancer care program in rural settings. We specifically focused on the factors related to specific needs of rural populations, care settings and resources. The CFIR is a framework particularly well-suited for implementation of multi-level and multi-component programs. The CFIR includes five domains (*intervention characteristics, outer setting, inner setting, characteristics of the individuals involved, and the process of implementation*), which are subsequently detailed into over 30 different constructs, or “sub-domains” that could serve as implementation barriers or facilitators. Where appropriate, we used publicly available data to assess relevant CFIR constructs (Supplementary Table 1) (3, 35–43).

### Data Collection and Analysis

Guided by the CFIR, we identified “individuals involved” (stakeholders) to include in the purposeful sampling for the qualitative data collection. The CFIR also guided the choice of themes/questions for the data collection addressing inner and outer settings and processes guiding decisions about cancer care delivery in rural settings. We used grounded theory method to then generate a process theory about rural cancer program implementation generated from the analysis of the data (44–46).

#### Qualitative Data Collection

Interviews were recorded and transcribed by the study coordinator and analyzed and interpreted by the V-ROC team. Four team members analyzed the data by using NVivo software (47) to perform inductive constant comparison. This approach involved systematically reading the transcribed interviews and focus group records, identifying themes and then proceeding to verify, confirm, qualify, and explain these themes by comparing data within and between participants. To ensure rigor and minimize coder bias, we reviewed and compared emerging codes and negative cases at weekly V-ROC team meetings.

We first collected qualitative data through focus groups with rural cancer care stakeholders to identify the most relevant factors and parameters affecting care delivery, planning and decision-making about cancer treatment and cancer programs implementation in the region. We used concurrent data generation and/or data collection and analysis so that results from the previous focus group were immediately incorporated in the discussion guide for the subsequent focus groups. We applied constant comparative analysis and iterative process to progress from the initial to intermediate to advanced (theoretical) coding.

##### Focus Groups

We conducted three focus groups, that included 43 participants and lasted 2 h 20 min total. The focus groups explored stakeholder values and preferences regarding disease and disability, attitude toward medical innovation, knowledge about cancer and cancer treatment, and resource availability that may distinguish care delivery, planning and decision-making about cancer treatment in rural vs. urban areas. Group 1 was a virtual cross-disciplinary focus group (*n* = 12, 90 min duration) that included physicians, nurses, cancer patients, and their caregivers. The participants were selected from the list of regional experts in oncology and rural primary care who have previously collaborated with the university medical center. The ThinkTank collaborative software (48) was used to screen-share, communicate virtually in real time, express preferences, confidentially rank participants' responses, and collect quantitative data from the participants of this focus group. Groups 2 and 3 were face-to-face focus groups with rural primary care physicians, surgeons, medical oncologists, oncology nurses, care managers, and practice administrators (group 2, *n* = 15, 30 min duration; and group 3, *n* = 16, 20 min duration). These focus groups were conducted during monthly regional care coordination committee meetings in one of the rural counties. All committee members were invited to participate in the focus groups, none refused.

##### Semi-structured Interviews

All focus group participants were invited to participate in semi-structured interviews. The final sample (*n* = 5) was determined using purposive sampling to further probe for sensitive or ambiguous themes that came up during the focus groups (e.g., patient-provider conflict, effect of financial incentives on provider behavior, role of publicly funded social programs). Interview questions were informed by the CFIR and focus group findings, but also included open-ended questions to encourage unbiased opinions (49). Guides for semi-structured interviews focused on the barriers highlighted by the focus group: individual barriers (awareness of cancer care guidelines for multidisciplinary care delivery, provider confidence in addressing current cancer care delivery limitations, provider beliefs/attitudes regarding their role in care coordination, and program implementation), regional and facility settings (access barriers and facilitators to care in rural communities, physician reimbursement, health IT capacity, and patient acceptability). Interviews with providers also included an assessment of practice finances and capacity, and knowledge about current standards for cancer care.

#### Quantitative Data Collection

Based on the initial qualitative findings, we developed surveys that were piloted with the initial group of respondents and then administered to a new group of rural stakeholders. The survey e assessed *the relative importance* of the individual and structural barriers to cancer care delivery and program implementation in rural communities and ranked *feasibility and acceptability* of potential solutions. Following each focus group, we collected data using web-based reporting tools (focus group 1) and paper questionnaires (groups 2–3) from all participants. In addition, the surveys were distributed to rural patients and caregivers at community free meal events (Group 4, *n* = 77). The questionnaires were based on the set of questions and priorities identified by the participants of focus group 1 and project key informants. Finally, we integrated both qualitative and quantitative findings by presenting the findings to a sub-group of rural stakeholders and discussing validity and possible interpretations of the findings (50).

Survey results were analyzed using SAS Software version 9.4 © SAS Institute Inc., Cary, NC, USA.

## Results

### Role of Provider Teams and Teamwork in Regional Cancer Care Delivery

Stakeholders across all categories reported that collaborations among healthcare, social and administrative services providers are essential for efficient and guideline-concordant cancer care delivery in rural counties.

“Having non-physician team members handle appointment scheduling requests, refill requests, form completion requests, etc. makes everything runs smoother…” (n1).

“Nurse would often triage a message and may well answer the patient's concern best, and fastest [compared to a physician]” (s6).

Participants also pointed out to a frequent discordance between provider's administrative titles (e.g., “county health department coordinator”) and their roles on patient care teams (e.g., patient navigator and liaison between community supportive services and cancer clinic). Both patients and providers identified this lack of clear roles and “rode maps” as an additional challenge for patients trying to identify and navigate available resources.

### Impact of Organizational Environment on Provider Functioning

One factor on which different stakeholders disagreed was the effect of provider reimbursement approach and practice organization on feasibility and effectiveness of cross-institutional cancer care teams. Providers who had experience working for large regional systems and in salaried environments were more optimistic about cross-institutional collaboration than individuals who worked in a fee-for-service solo provider environment.

“[Provider-to provider communication takes time and] needs to be incorporated into physician schedule—not something to be done between appointments. Quality metrics based on use [of teamwork] should also be tied into evaluation of physician performance” (s2).

“Reimbursement for physician time spent doing this important aspect of care (communicating and coordinating) is a real barrier” (m2).

### Perceptions and Attitude Toward Remote Care Delivery and Health IT

**Figure 1:**
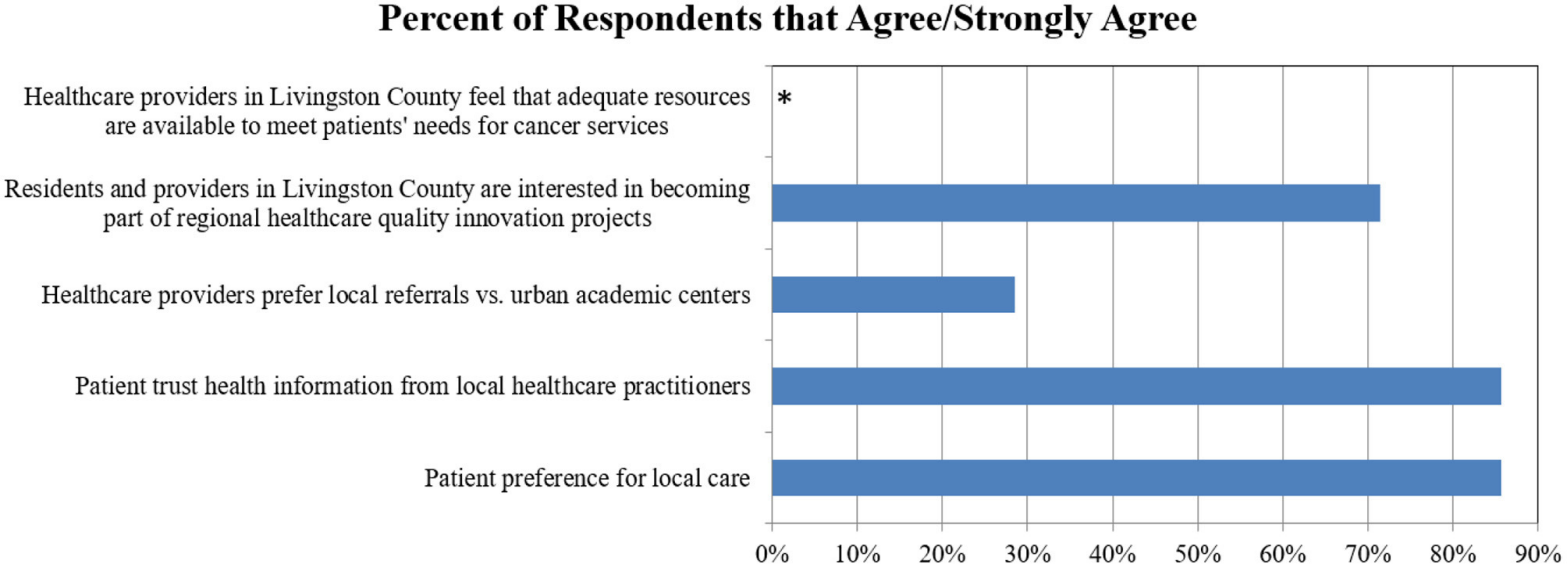
Provider preferences and attitudes for local and regional cancer services: percentage of respondents who Agree/Strongly Agree. *No healthcare providers agreed that adequate resources were available to meet patients' needs.

All stakeholders were open to using innovative health IT and telemedicine solutions to facilitate cross-regional care. However, no rural healthcare provider expressed confidence that adequate resources were available to meet patients' needs in rural settings (Figure 1).

“[Patient with] complex needs will often benefit from care manager input, so seems essential these persons would use [regional EMR application]” (s6).

However, both providers and patients pointed out to several barriers that may compromise quality and access to virtual cancer care in rural communities.

“Unreliable technology is often the main reason why people don't even try (communicating and coordinating)” (s6).

“Many patients don't have access to computer and computer literacy to make advantage of (remote communication with providers)” (p1).

“It sounds good in theory but what about technologic challenges for those not accustomed to this type of communication” (s6).

A number of concerns regarding variation in health insurance coverage, resources availability, supportive services, and professional expertise were also raised.

### Perception and Attitude Toward Implementation of New Cancer Care Programs

Participant responses revealed that organizational and individual capacity to support new program implementation varied by county and depended on other initiatives and challenges the stakeholders were facing at the time, including the role of opinion leaders (local economic, educational, spiritual, medical, and public health leadership), access to knowledge and information, available resources, relative priority, learning climate, as well as how the innovation was perceived by the key stakeholders in terms of the relative advantage it could provide, its complexity and costs.

“You need champions and advocates who will have true credibility—i.e., surgeons for other surgeons, GPs for other GPs, RNs for other RNs, etc.” (m2).

“Reimbursement for time spent in this work” (s6) were perceived extremely important both to demonstrate institutional commitment for innovation and to remove financial barriers for implementation that is significant for small rural practices with limited staff.

Respondents were also emphasized that non-financial incentives could be very effective for stimulating teamwork and cooperation including “patient satisfaction metrics” (s6) and “success stories from early adopters” (s6). From patient point of view, provider communication and encouragement were by far the most important motivation to adhere to new care protocols. As one patient put it “Definitely talk up advantages [of multidisciplinary care referrals] while patients are in for appointments with the medical team” (p2).

Reported threats to implementation of any new program included hospital, practices, and health systems mergers that could disrupt existing formal and informal networks and provider teams. Regional economic stability (e.g., factory or hospital closing) and public health threats (e.g., Zika or COVID-19 virus epidemic, opioid overdose crisis) redirected regional resources and stakeholder attention from what was perceived as low acuity problems (e.g., cancer survivorship). The summary of county resources and infrastructure in presented in Supplementary Table 1.

### Survey Results

Survey results demonstrated that while some rural healthcare providers emphasized the importance of local delivery of cancer services (28%), many more were open to regional cancer networks (72%) as long as their patients were appropriately supported locally (Figure 1). Most patients trusted recommendations they received from their local providers (86%) and preferred to receive care locally (86%) (Figure 1).

**Figure 2:**
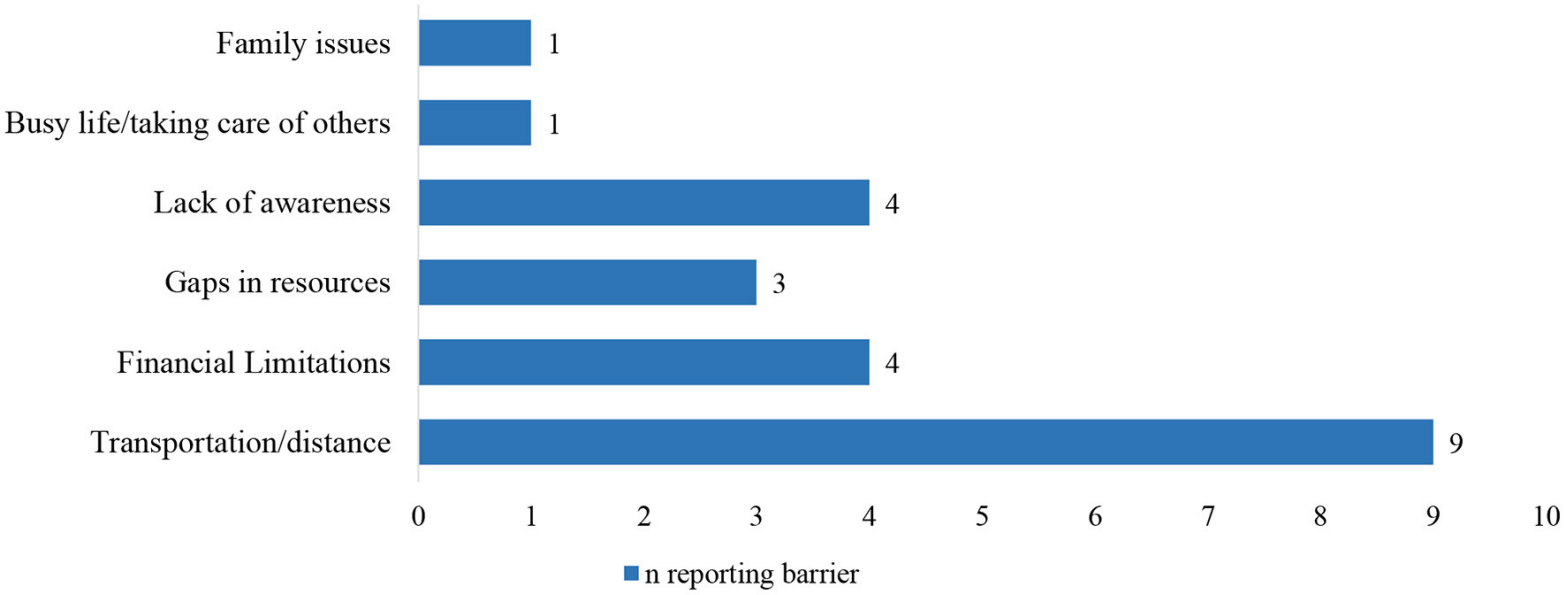
Reported barriers to cancer care for new patients and survivors in rural counties. Findings from Group 1, *n* = 12.

While distance to care was ranked as the most important barrier for access to regional cancer services, financial burden and lack of awareness about cancer treatment guidelines and local treatment options and resources were also important obstacles (Figure 2).

**Figure 3:**
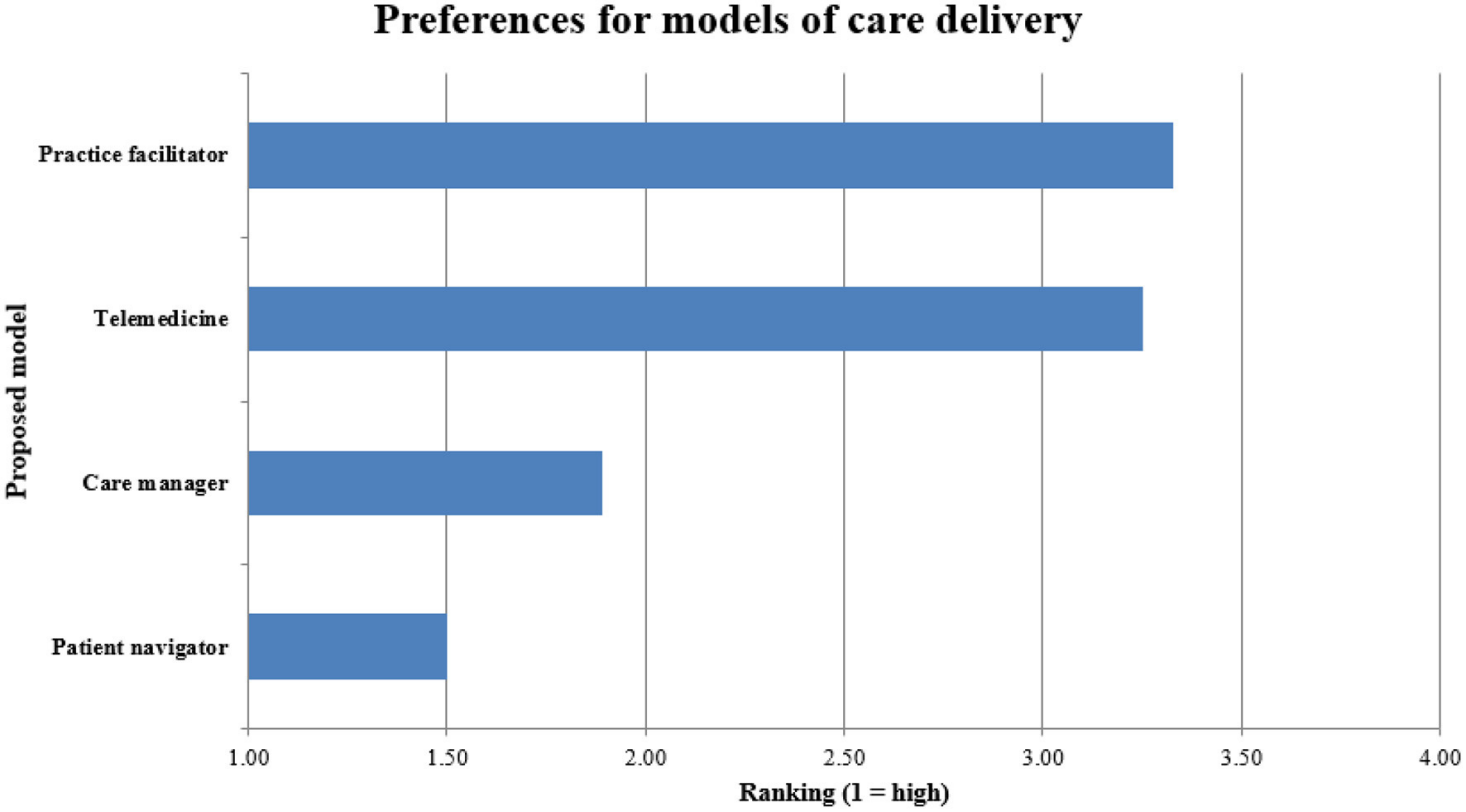
Rankings of different approaches for facilitating guideline-concordant cancer care delivery in rural communities. Findings from Groups 2–4, *n* = 108.

Respondents favored in-person, one-on-one care coordination such as patient navigators (average rank 1.5/5) or county care coordinators/managers (1.9/5) higher than telemedicine (3.3/5) and visiting practice facilitators (3.3/5) (Figure 3).

## Discussion

Current cancer care guidelines recommend cancer care that is multidisciplinary and collaborative, including cross-institutional and provider-to-provider collaborations (12, 16, 17, 51). The National Cancer Institute has long supported development and diffusion of team-based cancer care, including National Cancer Institute and ASCO initiatives such as the Teams in Cancer Care Delivery project in recent years (52). A multidisciplinary teamwork approach has been also recommended for non-cancer services in complex rural patients as well (53). Teamwork and trust among stakeholders have been identified as strong facilitators of successful implementation of evidence-based programs and interventions in various settings, especially in remote geographic areas (13, 54). Despite the evidence and recommendations, the majority of cancer patients in the US do not receive coordinated multidisciplinary team-based cancer care and that proportion is even greater among rural patients due to the complexity of rural cancer survivors' needs and scarcity of proximate resources (11, 55, 56).

Informal teams and teamwork have a long history in healthcare because they are an intuitive solution to care fragmentation, provider information burden and increasing specialization of medical care (57–59). Organizational psychologists define teams as two or more people who interact dynamically, interdependently, and adaptively to achieve a common goal (60–62). There is strong evidence that effective teamwork can improve patient and organizational outcomes for many health conditions including cancer and that team training in healthcare settings is effective (63–66). Despite this evidence and general support for healthcare teams, little effort is dedicated to promoting and supporting interdisciplinary teamwork in cancer care delivery (67).

Even with our small sample size in this pilot study, we identified three thematic structural dimensions affecting implementation of a guideline-concordant multidisciplinary cancer care programs in rural communities. These dimensions include (1) lack of integration among oncology, cancer supportive and primary care services within and across rural counties, (2) misalignment between clinical guideline recommendations and health insurance reimbursement and provider payment policies, and (3) regional variation in infrastructure and workforce availability that makes standard protocols difficult to follow.

**Figure 4:**
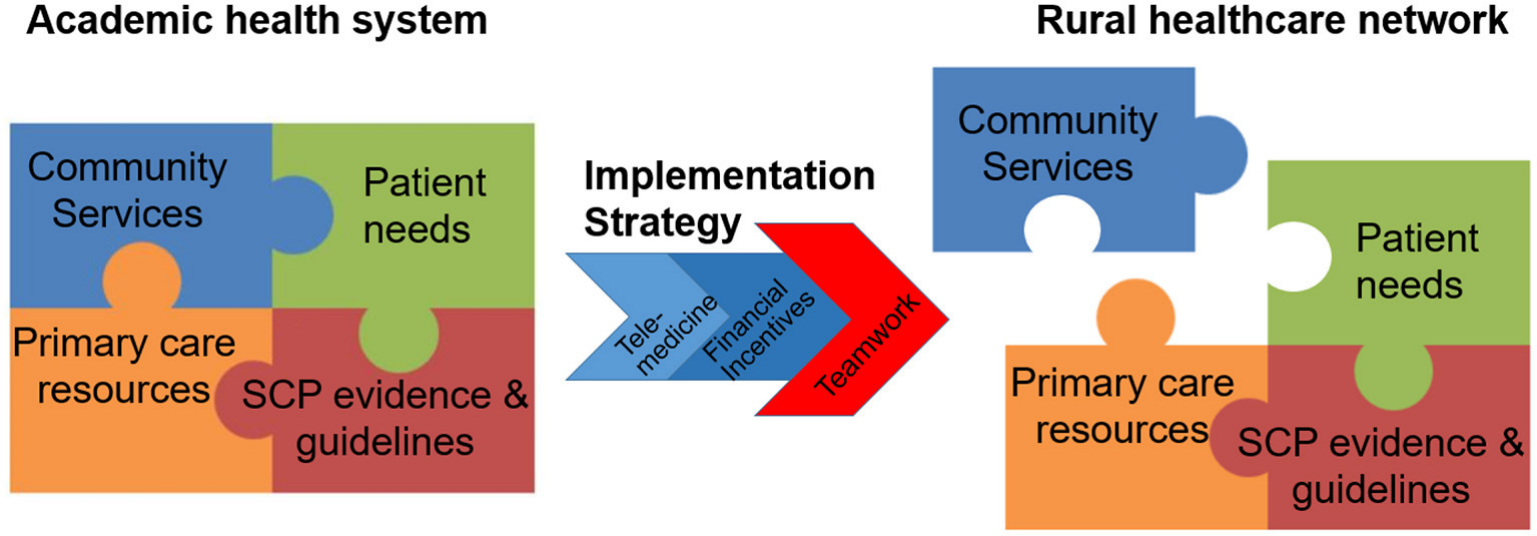
Using team science as a strategy to facilitate implementation.

Based on our findings, below we propose several strategies for planning and implementation of multidisciplinary cancer services programs in resource-poor communities that could facilitate adaptation of academic care delivery models for rural communities (Figure 4; Supplemental Table 2).

### Barriers to Care Integration Across Practices, Providers and Systems

Salas and colleagues demonstrated that team's efficiency depends on how well team members perform across seven specific domains of teamwork—team knowledge and attitude, cooperation, coordination, communication, cognition, coaching (feedback and monitoring) and identifying supportive conditions (timing, policy, alignment with larger organization priorities etc.) (64, 65, 68–73). In our study, provider stakeholders identified clinical teamwork as important facilitators for rural cancer care delivery even if they have not met other members of the “care team.” Attitudes toward virtual and remote teamwork were more positive among stakeholders who typically work in teams in their current job (e.g., nurses, social workers, public health department employees) compared to solo practitioners (e.g., physicians in solo practice).

Unlike many in-hospital or larger clinic-based teams where teamwork is dependent on face-to-face interaction, stakeholders in our study frequently described teams that consisted of several health providers who have never met face-to-face and were only connected through shared patients. Members of such “regional teams” often further separated by organizational boundaries, geographic distance, health insurance coverage (e.g., public community clinic vs. private practice), information systems, and privacy constraints (16).

Recent studies in cancer and other fields have provided evidence that quality care depends upon timely information exchanges and regular communication flow between all those stakeholders involved in treatment [including patients, specialist physicians, other specialty disciplines, primary care physicians (CPs), and support services] (74–76). Remote communication options such as Zoom meetings or regular conference calls between physicians have become more acceptable post- COVID-19 pandemic and may improve communication between physicians who do not routinely have an opportunity to de-brief in-person (77–79).

### Misalignment Between Patient-Centered Guideline Concordant Care Goals and Fee-for-Service Reimbursement

Multidisciplinary team-based models of cancer care delivery (e.g., tumor boards, survivorship care plans) were originally developed and implemented in large academic medical centers (80, 81). Large healthcare organizations in the United States often employ their providers (instead of contracting with them), and hence, could use financial and behavioral incentives to encourage desired provider behavior more efficiently (e.g., spending time in multidisciplinary tumor boards or on addressing patient social barriers to care) (82, 83). The academic large scale cancer care delivery model does not translate easily to rural settings where solo provider practices and fee-for-service reimbursement approach still dominate. Thus, an academic multidisciplinary cancer care model requires a significant adaptation to be sustainable and hence, acceptable, in community settings.

An additional challenge is that many supportive and highly effective cancer services that are in high demand among cancer patients are not billable or nor reimbursed by many health plans (e.g., consultations with social worker regarding financing options for out-of-pocket medical costs or about state/employer-specific disability policies, exercise, and nutrition counseling) (84). Finally, the existing reimbursement schedule does not offset the clinic staff time and efforts required to coordinate cancer care and assist patients with care navigation. Proposed amendments to the Social Security Act addressing payment models for cancer services have been introduced but have not moved forward (85, 86).

At large cancer centers, non-billable staff efforts could often be covered through other cost-sharing mechanisms, such as research, training or administration. In small rural clinics, funding opportunities for health providers are limited to billable services. Therefore, without a reimbursement model directly aligned with the realities of providing multidisciplinary integrated cancer care, such care is prohibitively expensive and unsustainable in rural settings.

### Regional Variation in Infrastructure and Workforce Availability

Our findings and prior research demonstrate that provider availability and care delivery patterns vary greatly among the rural counties (2). We observed significant variation in availability of providers (county hospitals, cancer, and primary care clinics), publicly funded cancer services programs, access to public transportation, and existence of local formal (e.g., provider collaborations) and informal (e.g., patient support) networks. Other studies have noted similar challenges that necessitate extensive tailoring and adaptation of evidence-based programs and care protocols to fit the needs of rural community practices and their patients (87). One potential solution to rural specialist shortages and long travel distances is provider tele-coaching via hub-and-spoke models (88–90). A hub-and-spoke model could serve as a platform to coach rural and remote area providers to assist with management of complex patients locally and improve care coordination across the region when referrals are necessary. Care navigation is a patient-centered approach that could help patients with making decisions about their care, better communicate with their providers and identify and more efficiently manage available local resources (91). However, care navigators are often employed by county health departments or social services agencies and are not usually disease-specific (92, 93).

The noteworthy strength of this study is its ability to collect information from a wide range of stakeholders, both rural and urban, cancer specialist and non-specialists, clinicians, and informal caregivers. As reported previously, recruitment of rural, low socio-economic status participants to studies can be time-consuming and challenging (94). Moreover, repeated data collection necessary for high quality statistical analysis represents an additional challenge to recruitment of special populations and may result in refusal to consent and high losses to follow-up (95, 96). Furthermore, because of low population density, the numbers of eligible cancer patients, by cancer type, in individual rural counties are too low for robust quantitative studies (97). To overcome these challenges, the authors draw on the principles of community-based participatory research in minority populations to identify a range of flexible, feasible and inclusive strategies that have been successfully used to recruit older people into clinical trials (30, 95). Despite the study's limited geographic scope, there was a wide variation in availability, attitudes and qualifications of health care provider ratios across several rural counties. Thus, the findings may be generalizable to a variety of rural settings. It is important to acknowledge that the study data collection took place in 2016–2017, before the seismic shift in the use of telemedicine precipitated by the COVID-19 pandemic. However, according to the recent study Chu et al. (98), the increase in the use of telemedicine in rural communities was significantly smaller than the corresponding change for urban patients. Chu's observations support the continuous importance of our findings for the field of rural cancer care delivery.

In summary, our findings revealed a unique combination of community, socio-economic, financial, and workforce barriers to implementation of guideline-concordant cancer care services in rural settings. One strategy to overcome these barriers is to improve provider cross-region communication and care coordination by means of team training and facilitation. Augmenting implementation planning with provider team-building strategies across and within regions could minimize implementation failure improve implementation efficiency, stakeholder buy-in, sustainability of guideline-concordant models of cancer care delivery, and continuity of care for cancer patients living in rural areas. Further research is needed to evaluate the effectiveness and cost-effectiveness of teamwork training and approaches as an implementation strategy in rural regions and other settings with limited specialist workforce availability.

## Data Availability Statement

The raw data supporting the conclusions of this article will be made available by the authors, without undue reservation.

## Ethics Statement

The University at Buffalo Institutional Review Board reviewed the study and made a Not Human Research determination because the study questions were focused on healthcare system and services delivery, not participant's health (UB IRB STUDY00004348). These accommodations were recommended by the study community champions to improve subject participation.

## Author Contributions

CC-I analyzed the study data, contributed to study conception, and interpreted the study results. NS, LC, and KN provided critical review, contributed to study conception, and interpretation. KN secured funding for the Patient-Centered Outcomes Research Institute (PCORI) Eugene Washington PCORI Engagement Award (2481-UB-IC E.N). PB contributed to study conceptualization, data collection, and manuscript preparation. FF, DH, IR, JR, LC, MS, and NT guided the development of stakeholder engagement plan and assisted with building the research network. KT helped identify IT platforms for the study data collection and developed the online patient and provider portal. All authors reviewed the paper, provided feedback on earlier drafts, and approved the final manuscript.

## Funding

This work was funded by a Patient-Centered Outcomes Research Institute (PCORI) Eugene Washington PCORI Engagement Award (2481-UB-IC E.N.). NS's research was supported by the National Institute for Health Research (NIHR) Applied Research Collaboration (ARC) South London at King's College Hospital NHS Foundation Trust. NS was a member of King's Improvement Science, which offers co-funding to the NIHR ARC South London. Its work was funded by King's Health Partners (Guy's and St Thomas' NHS Foundation Trust, King's College Hospital NHS Foundation Trust, King's College London and South London and Maudsley NHS Foundation Trust), Guy's and St Thomas' Charity, and the Maudsley Charity.

## Author Disclaimer

The views expressed in this publication are those of the authors and not necessarily those of the NIHR, the ESRC or the Department of Health and Social Care.

## Conflict of Interest

NS is the director of the London Safety and Training Solutions Ltd., which offers training in patient safety, implementation solutions, and human factors to healthcare organizations. LC reports royalties from UpToDate, Springer, Wolters-Kluwer, grant support from University of Alabama for Children's Oncology Survivorship Guidelines; all outside the submitted work. CC-I owns shares of Fortive Corporation and Danaher Corporation, outside of the submitted work. IR was employed by Mid-Atlantic Permanente Medical Group. NT was employed by Flatiron Healthcare Inc. KT was employed by KJT Group. The remaining authors declare that the research was conducted in the absence of any commercial or financial relationships that could be construed as a potentialconflict of interest.

## Publisher's Note

All claims expressed in this article are solely those of the authors and do not necessarily represent those of their affiliated organizations, or those of the publisher, the editors and the reviewers. Any product that may be evaluated in this article, or claim that may be made by its manufacturer, is not guaranteed or endorsed by the publisher.

## Abbreviations

CFIR: Consolidated Framework for Implementation Research
PCORI: Patient-Centered Outcomes Research Institute
V-ROC: Virtual Rural Oncology Community
PCOR: patient-centered outcomes research
APP: Advanced Practice Provider
IRB: Institutional Review Board
NYS: New York State
ASCO: American Society of Clinical Oncology
PCP: Primary Care Physician/Provider
ECHO: Extension for Community Healthcare Outcomes
NP: Nurse Practitioner
FTE: Full-time equivalent
PT: Physical Therapy
CT: Computerized Tomography.
