# Supplementary material for: Strategies for Improving Access to Cancer Services in Rural Communities: A Pre-implementation Study
Source: Front Health Serv. 2022 Mar 14;2:818519. doi: 10.3389/frhs.2022.818519 (PMC10012790; doi:10.3389/frhs.2022.818519)
Supplement: Supplementary file 1 [file Data_Sheet_1.docx]

*Supplemental Table A1: County-level characteristics*

| *County* | *Population*** | *Median household income*** | *% persons in poverty*** | *Population per square mile, 2010*** | *# farms*  *(% Δ since 2012)**** | *Farms with internet access (%)**** | *State rank by agricultural sales**** | *Population : PCP*  *ratio***** | *% uninsured***** |
| --- | --- | --- | --- | --- | --- | --- | --- | --- | --- |
| *Allegany* | *46,430* | *$45,359* | *16%* | *47.6* | *1* | *65%* | *31* | *2,480:1* | *7* |
| *Chemung* | *84,254* | *$51,251* | *15%* | *218.1* | *7* | *79%* | *48* | *1,310:1* | *5* |
| *Genesee* | *57,511* | *$54,033* | *11%* | *109.6* | *-12* | *78%* | *3* | *2,920:1* | *6* |
| *Livingston* | *63,227* | *$53,654* | *12%* | *103.5* | *-* | *83%* | *9* | *2,380:1* | *5* |
| *Orleans* | *40,612* | *$49,223* | *15%* | *109.6* | *2* | *72%* | *14* | *13,780:1* | *7* |
| *Schuyler* | *17,912* | *$47,810* | *14%* | *55.9* | *4* | *74%* | *39* | *1,390:1* | *6* |
| *Steuben* | *95,796* | *$50,157* | *13%* | *71.2* | *-7* | *77%* | *7* | *1,700:1* | *7* |
| *Wyoming* | *40,085* | *$55,459* | *11%* | *71.1* | *2* | *74%* | *1* | *2,400:1* | *5* |
| *Overall value* | ***445,827*** | ***$50,868*** | ***13%*** | ***98.3*** | *-0.4* | *75%* | *-* |  | *6* |
| *New York State* | ***19,542,209*** | ***$62,765*** | ***14%*** | ***411.2*** | *-* | *-* | *-* | *1,200:1* | *8* |

***Data from US Census(41)*

****Data from 2017 Census of Agriculture, population density < than 500 per sq mile is considered rural(39)*

*****County Health Rankings(43)*

*Supplemental Table A2: Integration of implementation science and teamwork frameworks to generate actionable findings*

| **Consolidated Framework for Implementation Research (CFIR)** | | | | | | |
| --- | --- | --- | --- | --- | --- | --- |
| - Networks and communications   External policies & incentives | - Implementation climate   Individual identification with organization | - Structural Characteristics | Individual identification with organization, self-efficacy, stage of change | Executing, Reflecting and evaluating, Engaging opinion leaders | Culture; Patient Characteristics; Campions | - Relative Advantage;   Cost; |
| **Effective Teamwork Principles** | | | | | | |
| Capability | Cooperation | Coordination | Communication | Cognition | Coaching | Conditions |
| ***Examples of strategies and approaches for rural settings*** | | | | | | |
| - Building networks and successfully communication real-time requires dedicated staff, which can be a challenge with stretched resources   Aim to identify key gatekeepers early (practice or community-based) | - Competing priorities may challenge implementation   Stakeholders’ perception of membership of a team increased participation in program activities | - Debriefing and soliciting stakeholder reflections in the end stages may help in implementation of future interventions | - Building on existing networks creates trusted relationships and faster dissemination | - Clear understanding among stakeholders regarding each group’s objectives and preferences fosters successful implementation | - A designated leader or leadership team is critical | - Models of delivery were developed for large academic centers for densely populated areas with adequate provider supply; tailored models for local staffing may be more successful in rural regions |
